# Chitosan-Based Polyelectrolyte Complex Cryogels with Elasticity, Toughness and Delivery of Curcumin Engineered by Polyions Pair and Cryostructuration Steps

**DOI:** 10.3390/gels8040240

**Published:** 2022-04-13

**Authors:** Ecaterina Stela Dragan, Maria Valentina Dinu, Claudiu Augustin Ghiorghita

**Affiliations:** Department of Functional Polymers, “Petru Poni” Institute of Macromolecular Chemistry, 700487 Iasi, Romania; vdinu@icmpp.ro (M.V.D.); claudiu.ghiorghita@icmpp.ro (C.A.G.)

**Keywords:** carboxymethyl cellulose, chitosan, poly(2-acrylamido-2-methyl-propansulfonic acid), polyelectrolyte complex, curcumin, drug delivery

## Abstract

Chitosan (CS)-based drug delivery systems (DDSs) are often stabilized by chemical cross-linking. A much more friendly approach to deliver drugs in a controlled manner is represented by polyelectrolyte complexes (PECs) physically stabilized by spontaneous interactions between CS and natural or synthetic biocompatible polyanions. PECs with tunable structures, morphologies, and mechanical properties were fabricated in this paper by an innovative and sustainable strategy. Carboxymethyl cellulose (CMC) or poly(2-acrylamido-2-methylpropanesulfonate sodium salt) were used as aqueous solutions, while CS microparticles were evenly dispersed in the polyanion solution, at pH 6.5, where CS was not soluble. Cryostructuration of the dispersion in two steps (5 min at −196 °C, and 24 h at −18 °C), and freeze-drying at −55 °C, 48 h, conducted to pre-PEC cryogels. Next step was rearrangement of complementary polyions and the complex formation inside the pore walls of cryogels by exposure of the pre-PECs at a source of H^+^. PEC cryogels with impressive elasticity and toughness were engineered in this study by multiple-cryostructuration steps using CMC as polyanion with a molar mass of 250 kDa and an optimum concentration of polyanion and polycation. The performances of PEC cryogels in sustained delivery of anti-inflammatory drugs such as curcumin were demonstrated.

## 1. Introduction

Bio-based drug delivery systems (DDSs) having polysaccharides (alginate, cellulose, chitosan, dextran, hyaluronic acid, carrageenan, starch) and/or proteins (casein, zein, lysozyme, soy protein) as building blocks attracted a large interest during the last decades due to their biocompatibility, accessibility, and biodegradability. Polysaccharides are preferred for the fabrication of DDSs due to their higher resistance to the environmental conditions (pH, enzymes, temperature) [1,2,3,4]. Among polysaccharides, chitosan (CS), the second biomass derived polysaccharide on the Earth after cellulose, the only cationic polysaccharide, obtained by the alkaline hydrolysis of the natural polysaccharide chitin, is used to prepare a large variety of nano/micro/macro-hydrogel 3D networks for targetable DDSs owing to its outstanding intrinsic features such as antifungal, antiviral, and antibacterial properties as well as biocompatibility and biodegradability, and the presence of reactive functional groups, such as –NH_2_ and –OH [1,2,3,4,5,6,7]. Because of the poor mechanical strength and high swelling ratio of many CS-based DDSs, these systems lead to burst release of drugs by breaking down the network. To overcome this issue, a combination between CS and a synthetic polymer such as poly(vinyl alcohol), widely used in biomedical and biochemical applications, seems to be a good choice [4]. The entrapment of aluminosilicates [8,9], or hydroxyapatite [10], into the CS network, with the formation of nanostructured biocomposite hydrogels, provided DDSs with improved delivery kinetics. Interpenetrating polymer network (IPN) hydrogels represent another safe route, which gives the possibility to modulate the properties of DDSs by generation of multi-stimuli-responsive networks [11,12,13,14]. In all these materials, chemical cross-linkers, more or less toxic for the human body, are usually employed.

Polyelectrolyte complexes (PECs) formed by spontaneous interactions between CS and natural or synthetic polyanions represent a more friendly approach to deliver drugs in a controlled manner [15,16,17]. Formation of PECs between two oppositely charged polyelectrolytes is well documented. At critical ratios between polyelectrolytes, aggregation occurs, and PECs are formed with the increase of the system entropy as a consequence of the release of a high number of small ions [18,19,20,21,22,23,24]. The main physical forces influencing PEC formation and their integrity are the electrostatic (Coulomb) interactions, van der Waals interactions, and hydrogen bonds. PEC properties could be tuned by the hydrophilic/hydrophobic balance of the complementary polymers, molar mass, charge density, ionic strength, and the mixing mode of polyions [24,25,26,27,28]. pH values are crucial in the case of PEC formation between weak polyelectrolytes [29,30]. PECs based on CS or related polymers have been used for the last decades in controlled release of drugs [31,32,33,34,35,36], controlled delivery of proteins and vaccines [37,38,39], and in dressings for wound healing [32,40].

**Figure 1:**
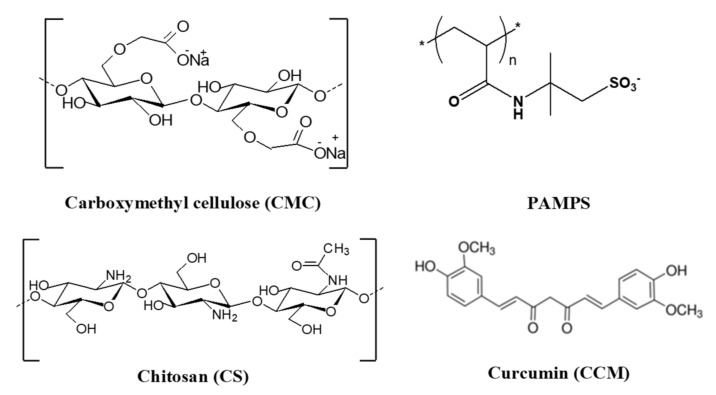
Molecular structures of polyelectrolytes used in the PECs formation and of curcumin used to test performances of PEC cryogels in controlled delivery of drugs.

Curcumin (CCM) is a natural polyphenol steadily investigated last decades due to its therapeutic effects such as anti-inflammatory, antioxidant, antimicrobial, anti-cancer (prostate, colon, breast), anti-aging, and potential anti-depressant properties [41,42,43,44]. The main limitation in using CCM for biomedical applications is its hydrophobicity, whose consequences are poor bioavailability, and deficient cellular uptake. To overcome these constraints, numerous CS-based delivery systems, responsive to internal or external stimuli, have been recently reported [45,46,47,48,49,50]. Oral delivery and slow release of CCM have been achieved by the encapsulation of CCM into quaternized aminated CS nanoparticles stabilized by ionic gelation using sodium tripolyphosphate as ionic cross-linker [46]. DDSs as nanoparticles, efficient as carrier for CCM, have been recently developed through the electrostatic interaction between CS and carboxymethylated corn fiber gum [49]. Porous bio-based biomaterials, owing to their outstanding characteristics such as uniform pore sizes, large inner surface areas, and pore volumes, represent sustainable materials, which demonstrated easy loading of drugs and offer numerous possibilities for controlled delivery of drugs, either for oral administration or for wound healing [7,8,50,51,52,53,54,55]. After cellulose, CS-based cryogels occupy a central place by their applications as DDSs, wound dressing, and tissue engineering [55,56,57], but only a few references are focused on PEC cryogels [57,58,59]. Therefore, one objective of this work is to develop novel CS-based PEC cryogels with morphology and elasticity controlled by the polyanion structure and molar mass as well as by the cryostructuration steps. The structures of polyelectrolytes used in this work for the fabrication of PECs as monoliths or cryobeads and of CCM are presented in Figure 1.

Our strategy for the preparation of PECs cryogels is based on multiple cryostructuration steps as follows: (i) CS powder was evenly dispersed in an aqueous solution of a biocompatible polyanion, which was either CMC, or PAMPS, a synthetic polyanion known for its biocompatibility [60,61]; (ii) pre-PEC cryogels were prepared by fast cryostructuration of the mixture first at −196 °C, for ~5 min, to freeze the homogeneous dispersion by unidirectional freezing, then at −18 °C for about 24 h; (iii) freeze-drying at −55 °C. To obtain PECs cryogels, the pre-PEC cryogels were exposed to a source of H^+^, for about 20 h. After washing, the cryogels were frozen at −18 °C, for 24 h, and finally freeze-dried. The as prepared PEC cryogels were characterized by mechanical properties such as elasticity and toughness. The influence of solution pH on the equilibrium water uptake (*WU_eq_*) was also investigated. The second objective of the study was to explore the performances of the newly fabricated CS-based PEC cryogels in the sustained release of CCM as a model of hydrophobic anti-inflammatory drugs. As far as we are aware, this type of PECs cryogels, with potential for controlled delivery of CCM, is reported for the first time in this work.

## 2. Results and Discussion

### 2.1. Preparation of CS-Based PEC Cryogels

Polyanion/CS sets used in the preparation of PECs developed in this work are presented in Table 1. It is well known that when complementary polyelectrolytes are used as aqueous solutions, there are two ideal mechanisms of PEC formation: “*ladder-like*”, when the opposite charges of the complementary polyelectrolytes are compensated in a strict order, and “*scrambled egg*”, characterized by an irregular compensation of charges [19]. Normally, the real mechanism is in between these two extremes because there are numerous factors which control the PEC formation, such as: the structure, molar mass, and concentration of the complementary polyelectrolytes, mixing mode, pH, and ionic strength.

**Figure 2:**
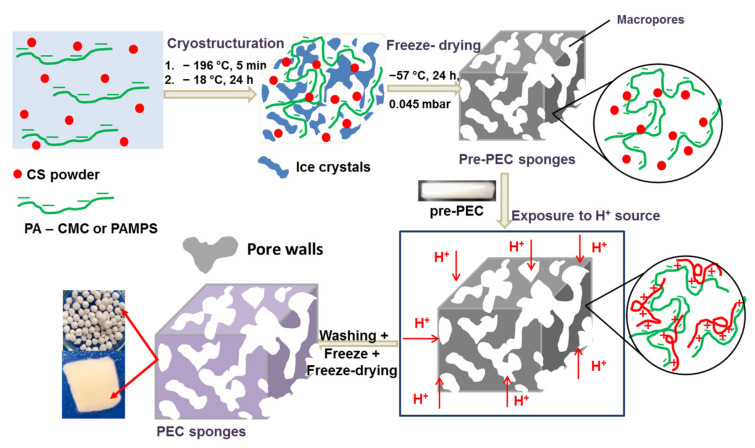
Main steps in the preparation of PEC sponges according to the strategy developed in this work.

According to the strategy presented in Figure 2, polyanions (CMC or PAMPS) were in the aqueous solution, while the polycation (CS) microparticles were evenly dispersed in the polyanion solution. By cryostructuration of the mixture at −196 °C, in liquid nitrogen (LN), the water molecules form ice crystals, the size of crystals decreasing with the speed of freezing [62,63,64,65], and with the decrease of temperature [66]. Fast freezing of the mixture at −196 °C, immediately after preparation, is essential to prevent changes in the homogeneous distribution of CS microparticles in the polyanion solution.

In the frozen system, polyanions and CS microparticles are concentrated in the pore walls, in a very dense phase, and forced to remain in this frozen arrangement even in the second step of cryostructuration at −18 °C. In the case of cryogel monoliths, the ice crystals grow along the longitudinal temperature gradient and the anisotropically ice crystals encompassed by the CMC walls containing CS microparticles are formed [54,62,65].

Freeze-drying the composite consisting of polyanion, CS microparticles and ice crystals at −55 °C preserve the distribution of polyanion chains and CS microparticles. Protonation of CS in the presence of a H^+^ source, in a closed environment, allows the PEC formation in a predetermined display of the two polyanions (CMC or PAMPS), on the one side, and CS, on the other side. The probability as this mechanism to be real increased with the increase of polyanion molar mass, the concentration, and the ratio between the complementary polyelectrolytes being also very important, as it will be seen later. As can be seen in Figure 2, the cryostructuration was repeated after the extraction of PEC monoliths or cryobeads.

### 2.2. Characterization of PEC Cryogels

#### 2.2.1. Structure, Morphology and Swelling

**Figure 3:**
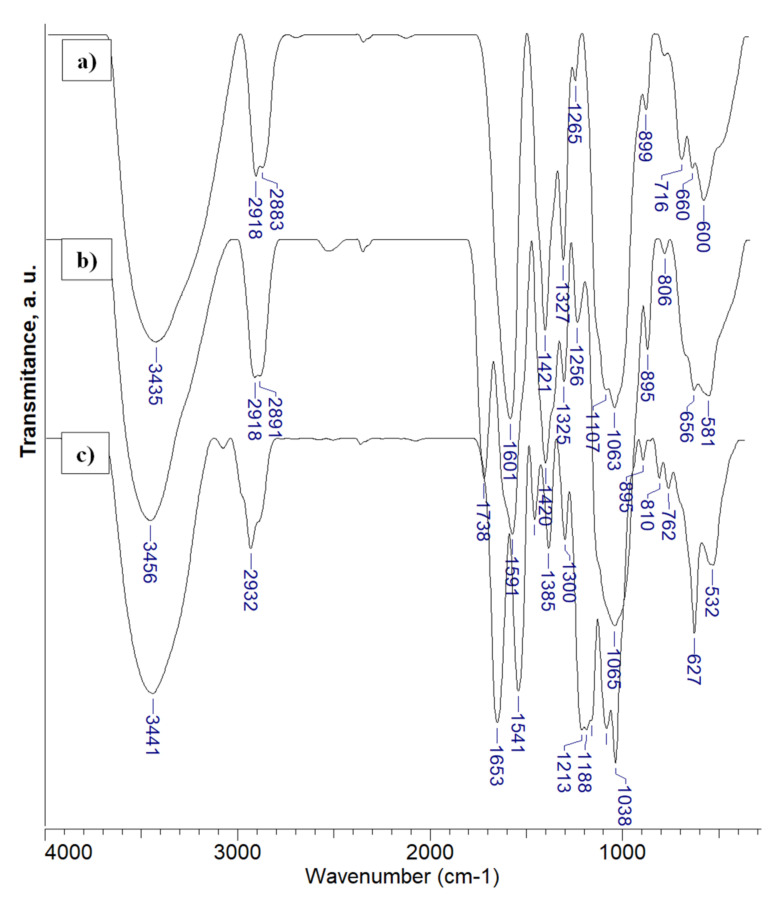
FTIR spectra of CS entrapped into the CMC cryogel (pre-2PEC.b, spectrum (**a**), 2PEC.b (spectrum (**b**), and PEC sample formed with PAMPS (3PEC.c, spectrum (**c**); the sample code is that allocated to the polyanion/CS sets in Table 1).

By FTIR spectroscopy, the main functional groups of pre-PEC and PEC cryogels were identified, as a function of the polyanion structure. The spectrum of pre-2PEC.b, where the CMC did not interact yet with CS (Figure 3), shows the presence of the following bands: a strong band at 3435 cm^−1^, attributed to O–H stretching, and inter- and intramolecular hydrogen bonds; two bands located at 2918 cm^−1^ and 2883 cm^−1^, ascribed to asymmetric and symmetric C–H stretching; two strong bands located at 1601 cm^−1^, and 1421 cm^−1^, assigned to the asymmetric and symmetric stretching vibrations of –COO^−^ functional groups; a small peak at 1265 cm^−1^ assigned to in plane bend of primary OH groups; the band located at 1327 cm^−1^, was assigned to the stretching vibrations of the C–N bond in the OC–N group in CS; the strong band at 1063 cm^−1^, and the peak at 899 cm^−1^ were assigned to the skeletal vibrations involving the C–O stretching in CS chains.

The FTIR spectrum of 2PEC.b supports the formation of PEC by the electrostatic interaction of CMC, negatively charged, with CS chains, which were protonated after the exposure to H^+^ source. Thus, the band characteristic to –COO^−^ functional groups diminished in intensity, being situated at 1591 cm^−1^, and a new band located at 1738 cm^−1^ arose due to the formation of some –COOH groups during the protonation of CS. The location of the other main bands was only slightly changed.

The FTIR spectrum of the 3PEC.c, taken as an example for the PAMPS/CS complexes, contains: a broad band at 3441 cm^−1^, assigned to N–H and O–H stretching, and inter- and intramolecular hydrogen bonds as well; a strong band at 2932 cm^−1^, attributed to C–H, CH_2_, and CH_3_ groups; an intense band at 1653 cm^-l^, given by the stretching vibrations of the C=O bond (amide I band); a strong band located at 1541 cm^−1^, assigned to the deformation vibrations of the N–H bond in secondary amide groups (amide II band); a small band located at 1458 cm^−1^, assigned to the deformation vibration of CH_2_ groups; a band of medium intensity situated at 1385 cm^−1^, assigned to C–H bonds; the small band at 1300 cm^−1^ was ascribed to the in plane C–N bending vibration [67]; a band at 1213 cm^−1^ arising from the O=S=O asymmetric stretching; the band characteristic to the SO_3_ asymmetric stretch, usually located at 1041 cm^−1^, is overlapped with the band at 1038 cm^−1^; the last main band situated at 624 cm^−1^ is attributed to the stretching of C–S bond. The presence of CS chains is supported by the peaks located at 1188 cm^–1^ (anti-symmetric stretching of the C–O–C bridge), the shoulder at 1080 cm^–1^, and the peak at 1038 cm^–1^ (skeletal vibrations involving the C–O stretching), which are characteristic to the polysaccharide structure, while the peak at 895 cm^−1^ was assigned to the wagging of the CS structure [67]. The FTIR spectra of the other PAMPS/CS complexes are similar with that presented in Figure 3. Figure S1 presents FTIR spectra for PECs prepared with CMC1 as cryobeads (1PEC.b) and as monolith with CS2 as polycation (2PEC.c). As can be observed, the main bands are located at about the same wavenumber as those of the sample 2PEC.b (Figure 3) and this supports the homogeneity of the samples prepared with the same polyanion (CMC).

**Figure 4:**
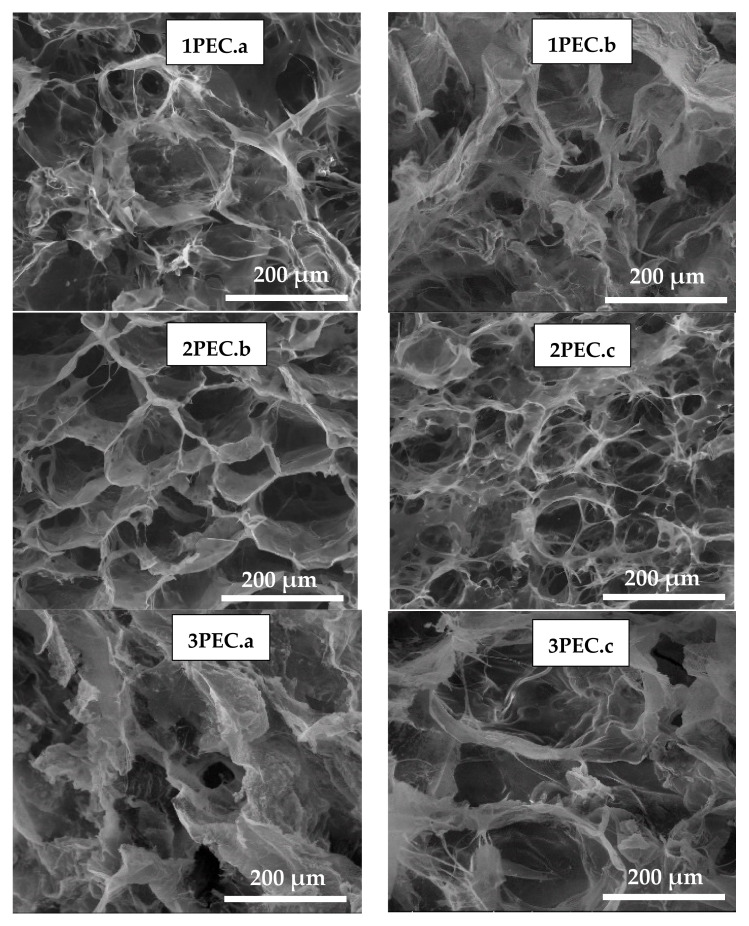
SEM images of PEC cryogels as a function of polyanion/CS sets (the sample code is that allocated to polyanion/CS sets in Table 1): mag. 500×, scaling bar 200 µm.

Figure 4 presents SEM images of polyanion/CS complexes as a function of polyanion molar mass and structure, as monoliths (1PEC.a, 2PEC.b, 2PEC.c, and 3PEC.a) or cryobeads (1PEC.b, and 3PEC.c). The honey-comb morphology with sizes of open pores in the range of tens of micrometers can be observed, the size and pore distribution depending on the CMC molar mass (1PEC.a compared with 2PEC.b). CS molar mass also has an influence on the PEC morphology (2PEC.b compared with 2PEC.c). The size of pores decreased with the increase of CS molar mass from 207 kDa to 305 kDa. The PEC morphology dramatically changed when PAMPS was used as polyanion (images 3PEC.a and 3PEC.c), the pores being larger and the pore sizes being more scattered. These features were assigned to the high flexibility of PAMPS chains, which could conduct to looser PEC morphologies.

Figure S2 presents the EDX spectra of the elements found on the surface of PECs. As can be seen, the element content was not influenced by CMC and CS molar mass, but by the structure of polyanion, the content of nitrogen being higher in the case of PAMPS as polyanion (complexes 3PEC.a, and 3PEC.c) than in the case of CMC. The presence of sulfur in a high amount supports the formation of PECs between CS and PAMPS.

**Figure 5:**
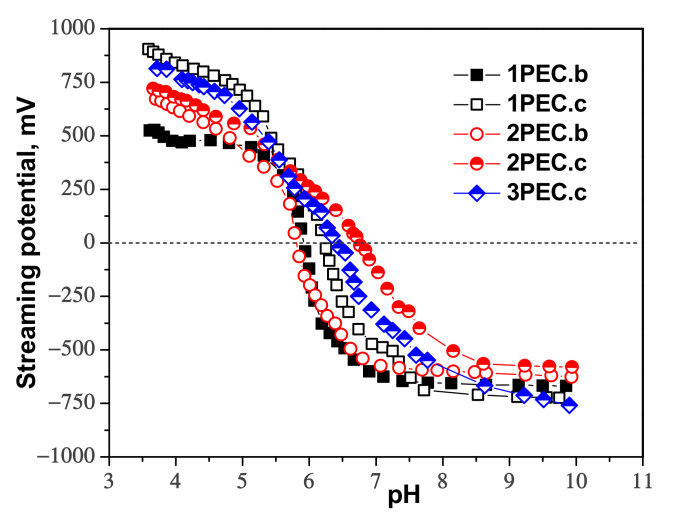
Streaming potential measurements of the PEC cryogels as a function of pH.

The pH where the point of zero charge (*pH_PZC_*) is located gives information about the free charges present on the surface of PECs particles as a function of pH. Figure 5 shows that the *pH_PZC_* for the complexes formed with CMC as polyanion is located in the range of pH 5.6–6.3, when CS1 was used as polycation, and at 6.7 in the case of CS2 (sample 2PEC.c).

These values demonstrate the compensation of opposite charges in a regular manner, even if the positive charges on the CS chains were created after the formation of the double ice-templated CMC cryogels. The increase of *pH_PZC_* when CS2 was used as polycation could be attributed to the decrease of CS chains flexibility and to the difficulties as the positive charges of CS to evenly interact with CMC chains in solid state, part of positive charges being extrinsic compensated (with counterions) [25]. In the case of the complexes formed with PAMPS (3PEC.c), the value of *pH_PZC_* was situated at around 6.3, i.e., at neutral pH. The explanation is associated with the high flexibility of PAMPS chains, which could much more easily interact with positively charged CS chains.

**Figure 6:**
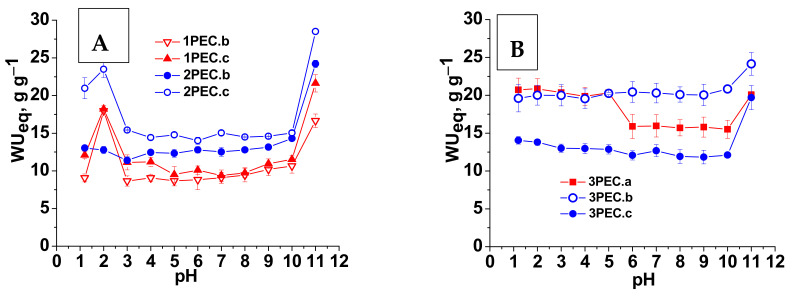
*WU_eq_* as a function of pH for PEC cryogels: (**A**) PECs with CMC as polyanion; (**B**) PECs with PAMPS as polyanion.

The response of a DDS to the medium pH is an essential characteristic when its performances in the delivery of a certain drug into the gastrointestinal (GI) tract are investigated. Therefore, the influence of pH on the equilibrium water uptake (*WU_eq_*) was explored in detail by the gravimetric method, the results being presented in Figure 6A (for PECs prepared with CMC as polyanion) and Figure 6B (for PECs prepared with PAMPS as polyanion). It seems that the absorption of water by these porous PECs is a very complex process. The absorption of water by the cryogel can be caused both by simple capillary suction and by the increase in the dielectric constant inside the cryogel leading to the decrease of the interaction energy between the –COO^−^ in CMC, or –SO_3_^–^ in PAMPS, and –NH_3_^+^. Furthermore, the swelling of PECs is facilitated by the osmotic pressure created by counterions, which move inside the cryogel but cannot leave it [58]. As can be seen in Figure 6A, the values of *WU_eq_* slowly increased with the increase of pH from 3.0 to 10, in the case of CMC1, an abrupt increase being observed for all PECs at pH 11. Most of the amino groups of CS are deprotonated at pH above 6.2. Increasing the pH from 7 to 10, the concentration of negatively charged groups in the PEC cryogel increases, and the polymer chains repel each other, and causes the values of *WU_eq_* to slowly increase. The abrupt increase of *WU_eq_* at pH 11, for all PEC cryogels, indicates the beginning of the complex destructuration occurred at this pH.

The PECs behavior at pH < 3.0 was strongly influenced by both the molar mass of CMC and CS (Figure 6A) and by the nature of anionic groups (PAMPS, Figure 6B). Thus, an abrupt increase of swelling was found for the PECs formed between CMC1 and CS1 (1PEC.b and 1PEC.c) as well as between CMC2 and CS2 (2PEC.c), with a maximum at pH 2 followed by an abrupt decrease at pH 1.2, which suggests starting of the complex erosion at this pH. On the other hand, the swelling of the complexes formed between CMC2 and CS1 monotonously increased up to pH 1.2, without a maximum at pH 2. This behavior supports the high stability of the complex formed in the last case. In the case of PAMPS/CS1 pair (Figure 6B), the swelling behavior as a function of pH, in the acidic range, was closer to that of complexes formed between CMC2 and CS1, with no visible maximum which also indicates the high stability of these complexes. A slight increase of the *WU_eq_* at pH 11 was observed also for these PECs, the values being lower than those observed in the case of CMC as polyanion. Optical images of the complexes 3PEC.a and 3PEC.b after their swelling in decreasing (acid medium) and increasing (basic medium) pH, presented in Figure S3, demonstrate the high stability of these complexes, which still preserve their shape at the end of the swelling cycles as a function of pH.

#### 2.2.2. Elasticity and Shape Memory Performance of PEC Cryogels

**Figure 7:**
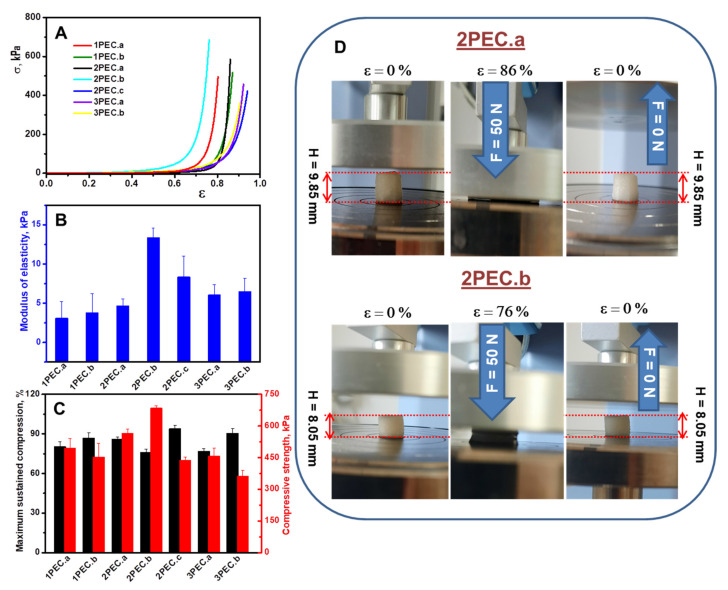
The mechanical properties of swollen PEC cryogels under compression: (**A**) stress-strain profiles of PEC cryogels; (**B**) Modulus of elasticity (blue color) determined according to the standard method; (**C**) Maximum sustained compression (black color) and compressive nominal stress (red color). Error bars represent standard deviation; (**D**) Optical images showing the compressibility, excellent elasticity, and instantaneous shape recovery of the 2PEC.a and 2PEC.b cryogels.

The uniaxial compressive tests were used to assess the mechanical stability of PEC cryogels. We were interested to establish the effect of polycation molar mass or polyanion structure as well as concentration and ratio between the complementary polyelectrolytes on the compressive mechanical performance of PEC cryogels. The compressive stress-strain (σ−ε) profiles of the PEC cryogels are presented in Figure 7A. All cryogels can be compressed to over 75% strain, without deformation or fracture of the gels at large deformation ratios, which is associated with the complete release of water from the honeycomb structures of PEC gels upon compression. Despite these similarities, the PEC cryogels demonstrated significantly different mechanical performance (elastic modulus, stiffness, and compressive nominal stress) depending on the molar mass of polyanion and polycation, the ratio between components, and the nature of polyanion (Figure 7B,C). Thus, by increasing the polyanion molar mass (1PEC.a compared with 2PEC.b), both the elastic modulus and the compressive nominal stress increased from 3.12 kPa and 494 kPa (sample 1PEC.a) to 13.37 kPa and 684 kPa (sample 2PEC.b), indicating the transition from an elastic network to a more dense and stiff one. On the other hand, by increasing the polycation molar mass (2PEC.b compared with 2PEC.c), a considerable improvement in the PEC flexibility was achieved; the 2PEC.c sustained 94% compression while the 2PEC.b sustained only 76.12% compression. However, 2PEC.b showed shape recovery, while 2PEC.c was irreversible deformed. The thin but dense pore walls of the 2PEC.b cryogels exhibit a remarkable stiffness and a high intrinsic mechanical toughness, which provides a great structural support to the entire highly interconnected porous matrix, and, thus, shape-memory performance (Figure 7D).

The elastic modulus and the compressive nominal stress decreased to 6.08 kPa and, respectively, 458 kPa when PAMPS was used as polyanion (3PEC.a), being correlated with the increase of the pore diameters and its water uptake. By increasing the PAMPS and CS concentration to 4 wt.%, a significant increase in the sustained compression to about 90% (sample 3PEC.b) was observed whereas the elastic modulus value remained almost the same as for sample 3PEC.a (with 3 wt.% concentration PAMPS and CS). Thus, the sustained compression of PEC cryogels could be modulated by controlling the polyanion and polycation concentration.

It should be pointed out the excellent mechanical properties of 2PEC.a and 2PEC.b cryogels (see Figure 7D), which after the load removal were capable of reabsorbing the water released during compression and recover almost completely their original shape. This indicates high elasticity, flexibility, non-brittleness, and a reversible behavior for these PEC cryogels. Furthermore, compared to other macroporous materials, our PEC cryogels displayed unexpectedly higher compressive strengths. Thus, 2PEC.b cryogel showed compressive stress value (at 76% strain) of 684 kPa, which was greater than 330 kPa (at 70% strain) for chitosan/sodium alginate PEC hydrogels [68], 133 kPa (at 80% strain) for polyacrylamide/poly(2-(dimethylamino) ethyl methacrylate)/cellulose nanocrystal/zinc oxide hybrid cryogels [69], 76 kPa (at 70% strain) for macroporous double-network cryogels based on polyacrylamide/poly(N-isopropylacrylamide) [70], 22.76 kPa (at 90% strain) for regenerated cellulose nanofiber reinforced chitosan hydrogel scaffolds [71]. In conclusion, it should be emphasized that PEC cryogels with remarkable elasticity and toughness were engineered in this study by multiple-cryostructuration steps using as polyanion CMC with a molar mass of 250 kDa and an optimum concentration of polyanion and CS of 3 wt.%.

### 2.3. Loading and Release of CCM in/from PEC Cryogels

It is known that the drug delivery kinetics in CS-based DDSs is depending on the route adopted for the drug administration, such as oral drug delivery, mucosal drug delivery, transdermal drug delivery, or parenteral [55,58,59]. The low-water soluble drugs, such as CCM, exhibit a limited in vivo drug dissolution leading to a low bioavailability of drugs [42,43,44,45,46,47,48,49]. Due to the pH-responsive feature of CS-based PECs, they could be suitable to control the drug release through a pH-dependent mechanism [55,58,59]. The loading and release of CCM in/from PEC cryogels were associated with the PEC structure and the average pore diameter evaluated from the SEM micrographs by the ImageJ 1.48v software (on three images, the number of pores measured per image being 15) [52,62]. As can be seen in Table 2, the highest loading with CCM was found in the case of PECs having CMC2 as polyanion, i.e., the PEC cryogels with the lowest pore diameter and the highest homogeneity of the pore distribution (see Figure 4). The loading with CCM of the PECs having PAMPS as polyanion (3PEC.a and 3PEC.b) was lower but comparable with that found in the case of the PECs prepared with CMC2.

The composition of release medium is essential when DDSs are engineered for oral administration. Therefore, some release media were tested before to decide on the most suitable one. The first release experiments of CCM from the PEC cryogels were performed from 2PEC.b and 2PEC.c, which have the same concentration of polyions and the same ratio between CMC and CS, with only the molar mass of CS being different (Table 1), with a mixture consisting of water:ethanol (80:20, *v*/*v*), at 37 °C. As can be seen in Figure S4, the release in pH 1.2 was fast in the first six hours, the maximum percentage of CCM released being ~12 wt.%, and 14.3 wt.%, from 2PEC.b, and 2PEC.c, respectively, which levelled off at ~13.87 wt.%, and 15 wt.%, after 22 h. Changing the release medium with PBS (pH 7.4) led to higher released amounts of CCM from both PECs, the increase being up to 15.25 wt.%, from 2PEC.b, and up to 19.6 wt.%, from 2PEC.c. The information, which this experiment brought to us, was as follows: the water:etanol (80:20, *v*/*v*) was not a suitable release medium for CCM; the molar mass of CS is an important parameter, which could be used to modulate the kinetics of drug release from the DDSs such as PECs cryogels.

**Figure 8:**
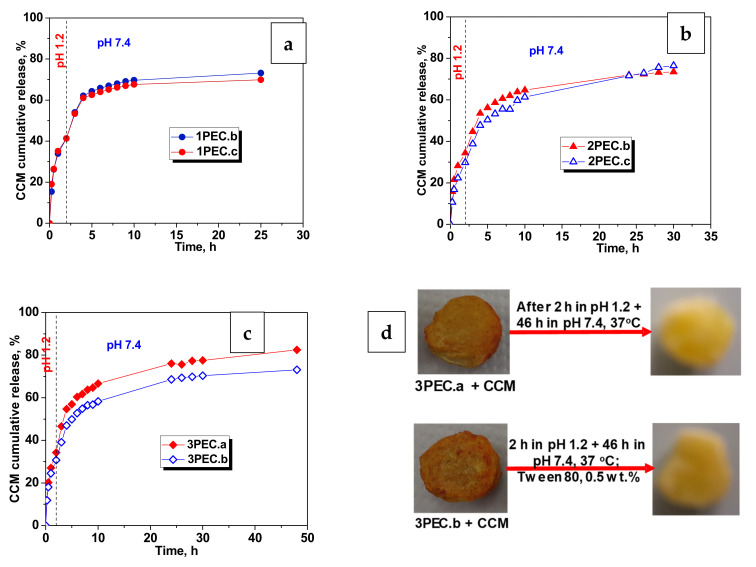
(**a**–**c**) CCM release kinetics in aqueous solution of Tween 80, 0.5 wt.%, at 37 °C; (**d**) optical images of PECs loaded with CCM and after partial release of CCM.

In the next series of experiments, aqueous solutions of Tween 80 with two concentrations (2 wt.% and 0.5 wt.%) were tested as release media of CCM from two PEC cryogels different only by their geometry: 1PEC.b, as monolith, and 1PEC.c, as cryobeads [48]. As displayed in Figure S5, the release kinetics were much faster than in the case of water:ethanol (80:20, *v*/*v*) (Figure S4), both at pH 1.2, and at pH 7.4, for both samples of PECs. The difference is consisting of the slower CCM release when the concentration of Tween 80 was 0.5 wt.%, the release being faster in the case of PEC monolith than from cryobeads. Therefore, the aqueous solution of Tween 80, with a concentration of 0.5 wt.% was chosen as release medium for further investigation of CCM release kinetics in sustained regime [46,47]. Using the aqueous solution of Tween 80, with a concentration of 0.5 wt.%, we simulated the passage of the designed PEC cryogels along the gastro-intestinal tract, first in pH 1.2 (simulated gastric fluid, SGF) for 2 h, then in pH 7.4 (simulated intestinal fluid, SIF) up to around 75–80% cumulative release of CCM. Figure 8 presents the sustained release profiles of CCM from 1PEC.b and 1PEC.c (Figure 8a), 2PEC.b and 2PEC.c (Figure 8b), and 3PEC.a and 3PEC.b (Figure 8c). All tested PEC cryogels exhibited a burst release of CCM in the initial 5 h, followed by a slow and sustained release afterwards. However, as can be observed in Figure 8, the composition of PEC cryogels influenced the CCM release profiles. Thus, the CCM release from the PECs prepared with CMC1 (Figure 8a) leveled off after 10 h, while the drug release from the PECs constructed with CMC2 went on up to 30 h and even more (Figure 8b). This fact shows the decisive role of the CMC molar mass and of the PEC morphology in the release kinetics. The presence of a flexible polyanion such as PAMPS in the structure of PECs had a strong influence on the CCM release kinetics (Figure 8c); the drug release was faster when PAMPS concentration was lower (3PEC.a compared with 3PEC.b). As can be seen, a sustained release of CCM occurred up to 48 h. The optical images of 3PEC.a and 3PEC.b loaded with CCM and after the partial release of CCM are displayed in Figure 8d, left and right, respectively. The presence of CCM in these complexes even after 48 h of the drug release demonstrate their performances in sustained release of drugs.

To investigate the release kinetics mechanism of CCM from PECs cryogels, the release data in Figure 8 were fitted by three kinetic models: first order kinetics (Equation (1)) [44], Higuchi model (Equation (2)) [72], and Korsmeyer–Peppas model (Equation (3)) [73], and these equations are presented below:

(1) $$M_{t}=M_{0}-exp^{-k1\cdot t}$$

(2) $$M_{t}=k_{H}t^{1/2}$$

(3) $$\frac{M_{t}}{M_{\infty }}=k_{KP}\;t^{nr}$$

where: *k*_1_ the constant for first-order model; *k_H_* is the Higuchi constant; *M_t_* and *M_∞_* are the cumulative amounts of CCM released at time *t* and the maximum released amount (released at infinite time); *k_KP_* is a constant related to the matrix; *n_r_* is diffusional exponent that gives indication about the release mechanism; *M_o_* is the initial amount of drug.

**Figure 9:**
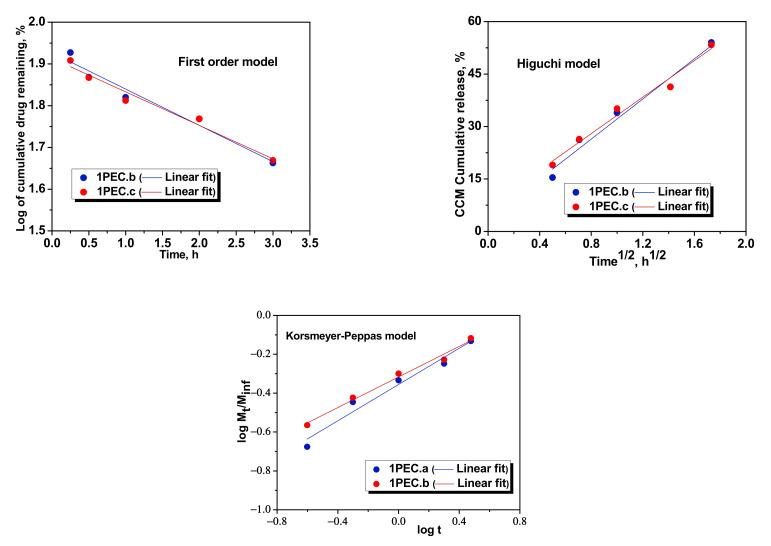
Fitting of kinetic models for the CCM release from 1PEC.b and 1PEC.c cryogels.

As can be seen in Figure 9, a satisfactory linear relationship was exhibited for all the three kinetic equations fitted on the experimental kinetics in the case of CCM release from 1PEC.b and 1PEC.c.

**Figure 10:**
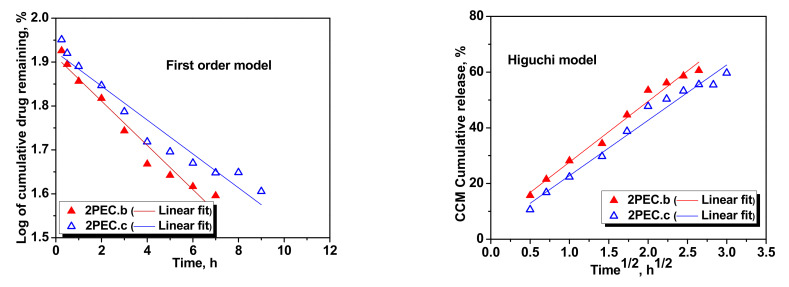

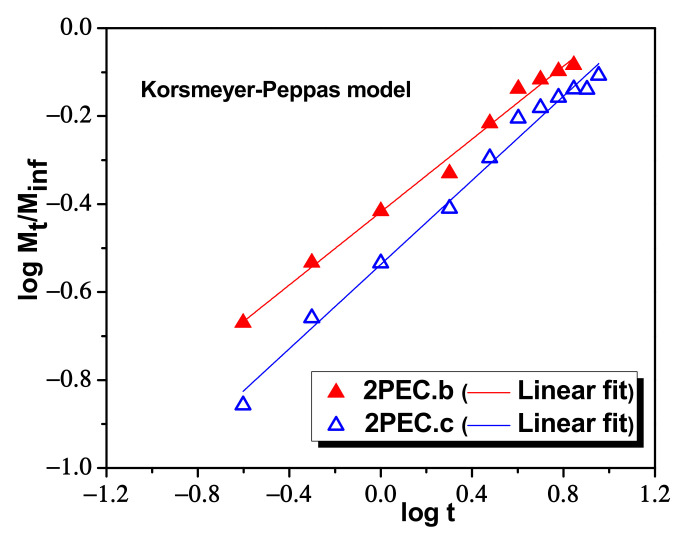
Fitting of kinetic models for the CCM release from 2PEC.b and 2PEC.c cryogels.

The kinetic models were fitted also on the CCM release data from 2PEC.b and 2PEC.c cryogels, and the results are presented in Figure 10.

**Figure 11:**
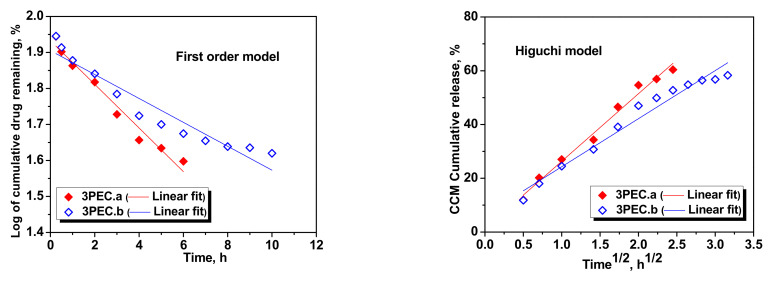

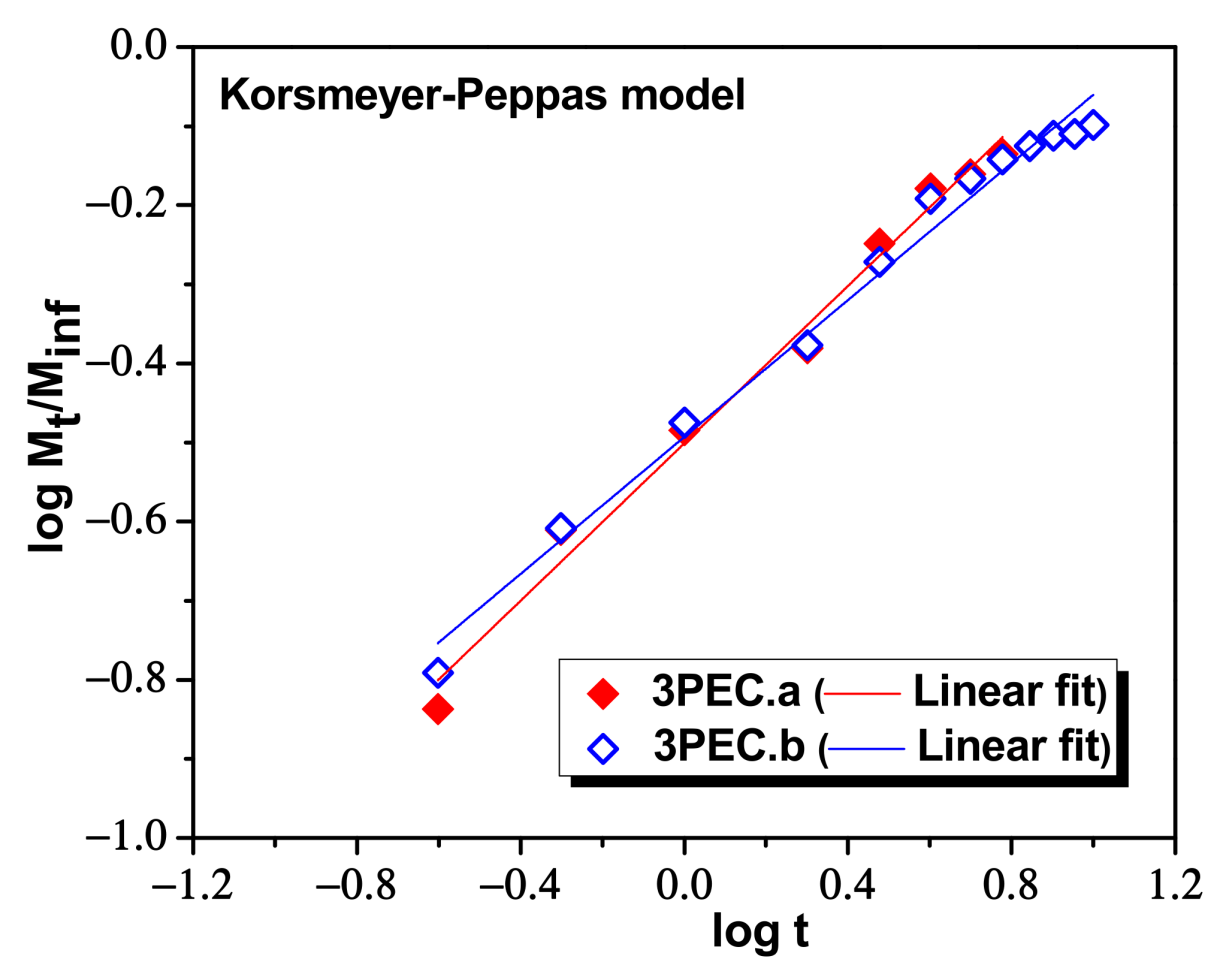
Fitting of kinetic models for the CCM release from 3PEC.a and 3PEC.b cryogels.

It is obvious that in this case, the first order kinetic model did not fit well the experimental release data, while the Higuchi and Korsmeyer–Peppas models described well the kinetics, with the *R*^2^ values in the range 0.975 to 0.99. From Figure 11, it can be observed that the first order kinetic model did not fit well the experimental release data for the 3PEC.b cryogel (*R*^2^ = 0.915), but the Higuchi and the Korsmeyer–Peppas models gave a good linear relationship for both 3PEC.a and 3PEC.b cryogels.

The kinetic parameters and the values of the coefficient of determination, *R*^2^, are presented in Table 3.

As displayed in Table 2, all the values of *n_r_* in the Korsmeyer–Peppas model are <0.5, and this support the Fickian diffusion controlled release mechanism of CCM from the PECs cryogels [57,73].

## 3. Conclusions

The chemical structure and physical morphology and elasticity of the PEC cryogels were tuned in this work by the structure, molar mass, and concentration of the anionic polyelectrolyte, and the CS molar mass. The structure and morphology of PEC cryogels were assessed by FTIR and EDX spectroscopy and SEM, respectively. Swelling of the PEC cryogels as a function of pH gave valuable information about their potential as promising systems for sustained release of drugs. It should be stressed that PEC cryogels with remarkable elasticity and toughness were engineered in this study by multiple-cryostructuration steps using CMC as polyanion with a molar mass of 250 kDa and an optimum concentration of polyanion and polycation. The release of CCM, taken as a model anti-inflammatory drug, from various PECs cryogels, in Tween 80 (0.5 wt.%), in SGF (2 h), and SIF (up to 46 h) were investigated. The release kinetics were fitted with three kinetic models (first order kinetic model, Higuchi model, and Korsmeyer–Peppas model). It was found that the values of *n_r_* in the Korsmeyer–Peppas model were <0.5 for all tested cryogels and this supports the Fickian diffusion controlled release mechanism of CCM from these materials. Thus, this study is oriented on a yet hot area of research, which is that of CS-based PEC cryogels with promising performances in sustained release of hydrophobic anti-inflammatory drugs, such as CCM, for oral administration.

## 4. Materials and Methods

Chitosan (CS) with molar masses of 207 kDa (CS1) and 305 kDa (CS2), and CMC with molar mass of 90 kDa (CMC1) and 250 kDa (CMC2), purchased from Sigma Aldrich, were also used as received. Curcumin (CCM) (96%) and PBS purchased from Sigma Aldrich were used as received. PAMPS with a molar mass of 1400 kDa was synthesized according to the method presented elsewhere [21]. Acetic acid, HCl, and NaOH were purchased from Chemical Company (Romania) and used as received. Molar mass of CS was determined as previously shown [74]. Deacetylation degree (DA) determined by FTIR was about 85%. Molar mass of PAMPS was determined according to the method previously presented [75].

### 4.1. Preparation of PEC Cryogels

PECs with different structures and geometries were prepared in this work as follows: (i) polyanion aqueous solution with a certain concentration was prepared first and used after 24 h; (ii) a certain amount of CS powder (see Table 1) was well dispersed in 10 g of polyanion aqueous solution, under a vigorous magnetic stirring (700–800 rpm), and kept under stirring 2 h, at room temperature; (iii) the homogeneous dispersion was either loaded in two syringes of 5 mL, closed with parafilm, and unidirectional frozen at −196 °C for ~5 min to get monoliths, or dropped into liquid nitrogen (−196 °C) to get cryobeads [76,77], and then transferred into a cryostat at −18 °C, for 24 h; (iv) after 24 h, the syringes with monolith cryogels were kept at room temperature about 5 min, and then cut into fragments of about 10 mm length, and immediately transferred into a Martin Christ, ALPHA 1-2LD device, for freeze drying (48 h, at −57 °C and 0.045 mbar); cryobeads were also freeze-dried in the same conditions; (v) after that, all samples were transferred into a closed environment containing a source of H^+^ (acetic acid) [78], and kept 24 h, as the PECs to be formed. The physically cross-linked composite sponges were washed with distilled water, 48 h at least, to remove any soluble components, and freeze-dried again as mentioned above.

### 4.2. Characterization of PEC Cryogels

The functional groups contained by the pre-PEC and PECs cryogels was investigated by FTIR spectroscopy with a Bruker Vertex FTIR spectrometer (Bruker, Ettlingen, Germany), resolution of 2 cm^−1^, by KBr pellet technique, with 5 mg composite. The samples were scanned in the range of 4000–400 cm^−1^. The interior morphology of the composite cryogels was explored by SEM using an Environmental Scanning Electron Microscope (ESEM) (FEI Company, Hillsboro, OR, USA) type Quanta 200, under vacuum, at 20 kV, with secondary electrons, coupled with EDX (SEM-EDX) for determination of the elemental composition. The mechanical tests were carried out on swollen cryogels, as monoliths of about 10–12 mm in diameter and 7–10 mm length, at room temperature, using a Shimadzu Testing Machine (EZ-LX/EZ-SX Series, Kyoto, Japan). A complete contact between the surface of cryogels and the compression plates of the testing machine was ensured by applying an initial force of 0.1 N before performing each analysis. The compressive strain (ε), stress (σ, kPa), and the elastic moduli (G, kPa) were evaluated according to the previously published protocol [79].

The evaluation of the swelling at equilibrium as a function of pH was performed by immersing the PEC samples in water of certain pH for 8 h, and after that the samples were weighed after wiping the excess surface liquid by filter paper. The *WU_eq_* (g/g) was calculated by Equation (4):

(4) $$WU_{eq}=\left(W_{eq}-W_{d}\right)/W_{d}$$

where: *W_eq_* is the weight (g) of the hydrated cryogel et equilibrium, and *W_d_* is the weight (g) of the dried cryogel.

Potentiometric titrations were performed using a PCD-03 particle charge detector (PCD 03; Mütek GmbH, Germany) to determine the *pH_PZC_* values of the PECs, defined as the pH where the streaming potential is zero mV. They were carried out between pH ≈ 3.5 and ≈ 10 by adjusting the pH of an aqueous suspension of microparticles using 0.1 mol/L HCl and NaOH, respectively.

### 4.3. Loading and Release of CCM from PEC Cryogels

Composite cryogels were loaded with CCM by the sorption-solvent evaporation technique [80]. Solutions of CCM in ethanol with a concentration of 5 mg/mL were prepared first and added to certain amounts of cryogels as carriers up to the maximum sorption capacity. The samples were kept 24 h in closed bottles, at +4 °C, in the dark, for the equilibration of drug sorption. After that, the bottles were opened and kept 24 h in the dark for solvent evaporation, and then transferred into the vacuum oven, in the dark, for 48 h. The loading of PEC cryogels with CCM was evaluated by weighing the dried samples (data presented in Table 2).

The in vitro release of CCM was performed in SGF, at pH 1.2, by immersing the sample loaded with CCM in 10 mL release medium containing 0.5 wt.% of Tween 80, if other concentration was not specified. At predetermined time intervals, 1 mL of supernatant were withdrawn and analyzed for the concentration of CCM at λ_max_ of 431 nm using a UV-Vis Spectrophotometer (SPECORD 200 Analytik Jena), based on a previously made calibration curve. The removed solution was replaced with an identical volume of fresh releasing solution to keep the volume constant. The cumulative release of CCM was calculated using Equation (5):

(5) $$CM\;\left(released\right)=\left[\frac{10C_{n}+\sum C_{n-1}}{m_{o}}\right]\times 100$$

where: *C_n_* and *C_n_*_−1_ are the concentrations of CCM (mg L^−1^) in the releasing medium after *n* and *n*^−1^ withdrawing steps; *n* is the number of withdrawing steps of the release medium; *m_o_* is the amount of drug loaded in the sample.

## Figures and Tables

**Table 1:** Polyanion/CS sets used in the fabrication of PEC cryogels.

Sample ID	Polyanion Solution			Chitosan			PEC Shape *
	Name	M_v_, kDa	Conc., wt.%	Name	M_v_, kDa	wt.%	
1PEC.a	CMC1	90	3	CS1	207	3	UF
1PEC.b	CMC1	90	4	CS1	207	4	UF
1PEC.c	CMC1	90	4	CS1	207	4	CB
2PEC.a	CMC2	250	3	CS1	207	3	CG
2PEC.b	CMC2	250	3	CS1	207	3	UF
2PEC.c	CMC2	250	3	CS2	305	3	UF
3PEC.a	PAMPS	1400	3	CS1	207	3	UF
3PEC.b	PAMPS	1400	4	CS1	207	4	UF
3PEC.c	PAMPS	1400	4	CS1	207	4	CB

* UF—unidirectional freezing; CB—cryobeads; CG—conventional cryogel.

**Table 2:** Pore diameter and drug loading of PEC cryogels selected for the investigation of CCM release.

Sample Code	1PEC.b	1PEC.c	2PEC.b	2PEC.c	3PEC.a	3PEC.b
Pore diameter, µm	126.5 ± 11	109.5 ± 38	107.9 ± 21.5	51.7 ± 11	117.8 ± 18	107.8 ± 28
Drug loading, %	6.67	5.62	8.9	9.73	8	7.7

**Table 3:** Kinetic parameters for the release of CCM from PEC cryogels.

Model Name	Parameters	PEC Samples					
		1PEC.b	1PEC.c	2PEC.b	2PEC.c	3PEC.a	3PECb
First order	*k*_1_	−0.0869	−0.0803	−0.0504	−0.0386	−0.0606	−0.0332
	*R* ^2^	0.9549	0.9634	0.9584	0.9379	0.9679	0.9150
Higuchi	*k_H_*	28.6841	26.0885	21.8286	19.8796	25.1502	17.8917
	*R* ^2^	0.9644	0.9742	0.9826	0.9753	0.9848	0.9657
Korsmeyer–Peppas	*n_r_*	0.4663	0.3934	0.4146	0.4788	0.4974	0.4327
	*k_KP_* (min^−nr^)	7.0075	7.2855	6.5836	5.8426	6.0586	6.1079
	*R* ^2^	09563	0.9824	0.9911	0.9886	0.9858	0.9859

## Data Availability

Not applicable here.

## Supplementary Materials

The following supporting information can be downloaded at: https://www.mdpi.com/article/10.3390/gels8040240/s1, Figure S1: FTIR spectra of PECs based on CMC1 and CS1 polyions pair (1PEC.c), and CMC2 and CS2 pair (2PEC.c); Figure S2: EDX spectra of PEC cryogels as a function of the polyanion: CS sets (see Table 1); Figure S3: Optical images of two PECs prepared with PAMPS as polyanion, at the end of the cycle of swelling in acid medium (left), the last pH is 1.2, and at the end of the cycle of swelling in basic medium (right), the last pH is 11.0 (samples are connected with Figure 6b); Figure S4: Cumulative release of CCM from the samples 2PEC.b and 2PEC.c (the same abbreviation as in Table 1). Loading of the samples was: 89 mg CCM/g 2PEC.b and 97.3 mg CCM/g 2PEC.c; Figure S5: Cumulative release of CCM from the samples 1PEC.b and 1PEC.c. Loading of the samples was: 66.74 mg CCM/g 1PEC.b and 56.25 mg CCM/g 1PEC.c.
