# Supplementary material for: Chitosan-Based Polyelectrolyte Complex Cryogels with Elasticity, Toughness and Delivery of Curcumin Engineered by Polyions Pair and Cryostructuration Steps
Source: Gels. 2022 Apr 13;8(4):240. doi: 10.3390/gels8040240 (PMC9024878; doi:10.3390/gels8040240)
Supplement: Supplementary file 1 [file gels-08-00240-s001.zip › gels-1682594-supplementary.pdf]

gels

Supplementary Materials

Chitosan-Based Polyelectrolyte Complex Cryogels with Elasticity, Toughness and Delivery of Curcumin Engineered by Polyions Pair and Cryostructuration Steps

Ecaterina Stela Dragan *, Maria Valentina Dinu and Claudiu Augustin Ghiorghita

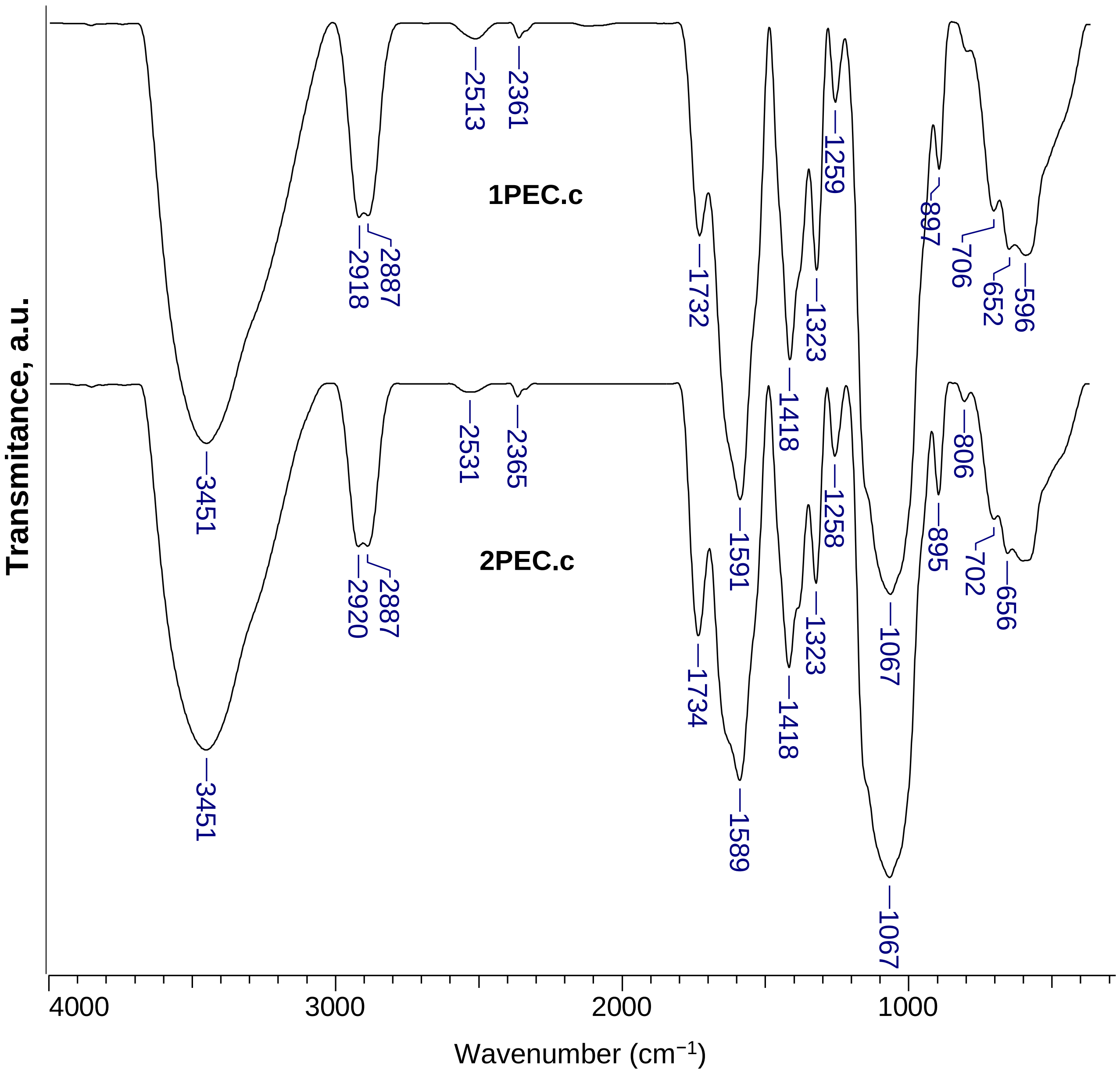

Figure S1. FTIR spectra PECs based on CMC1 and CS1 polyions pair (1PEC.c), and CMC2 and CS2 pair (2PEC.c).

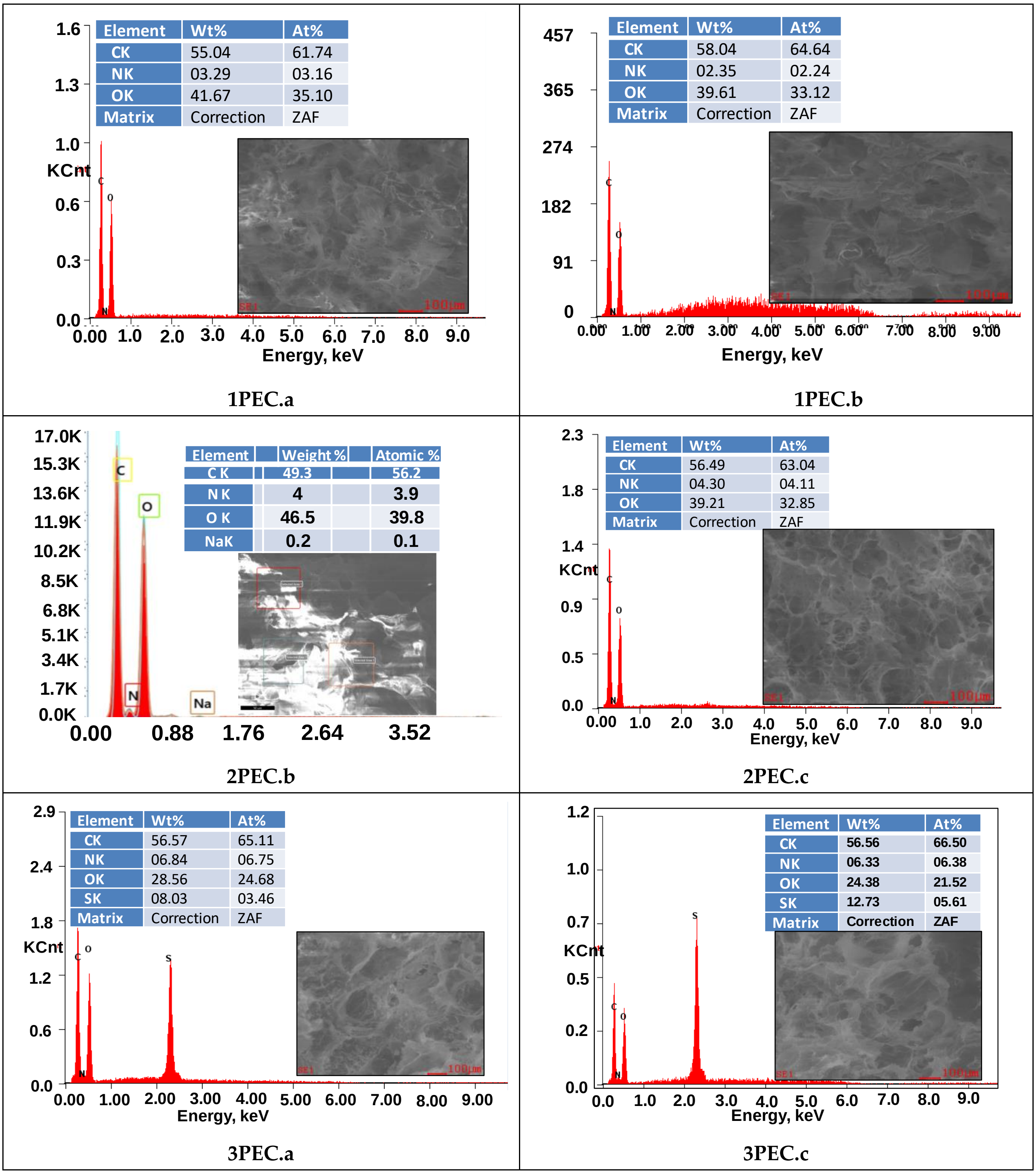

Figure S2. EDX spectra of PEC cryogels as a function of the polyanion : CS sets (see Table 1).

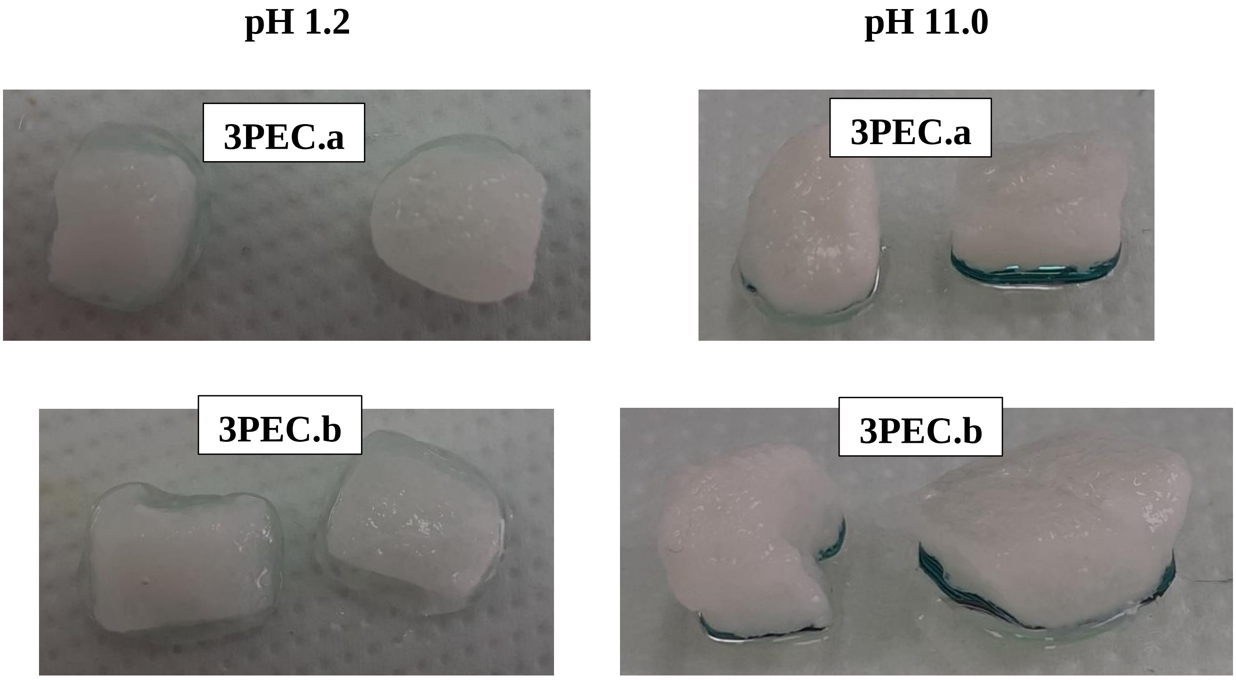

Figure S3. Optical images of two PECs prepared with PAMPS as polyanion, at the end of the cycle of swelling in acid medium (left), the last pH is 1.2, and at the end of the cycle of swelling in basic medium (right), the last pH is 11.0 (samples are connected with Figure 6b).

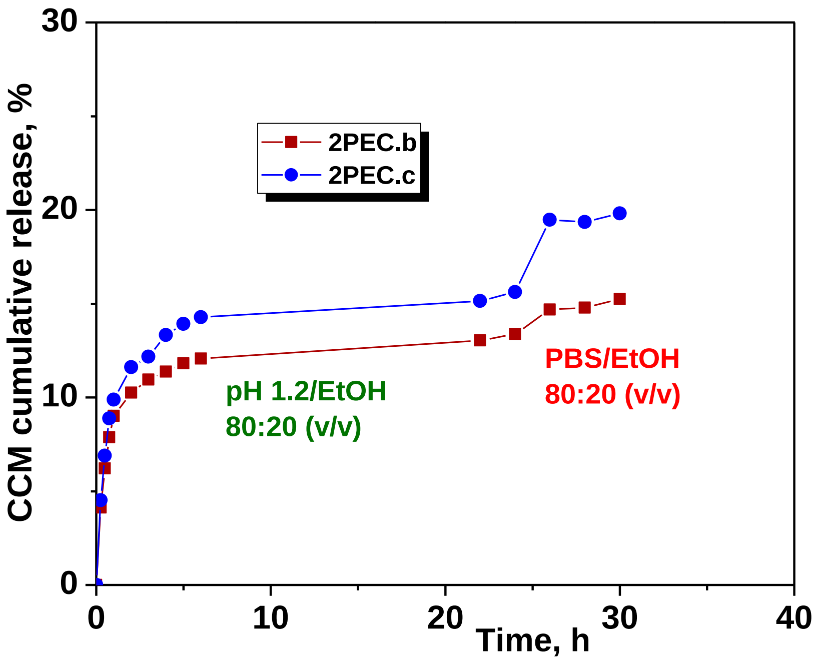

Figure S4. Cumulative release of CCM from the samples 2PEC.b and 2PEC.c (the same abbreviation as in Table 1). Loading of the samples was: 89 mg CCM/g 2PEC.b and 97.3 mg CCM/g 2PEC.c.

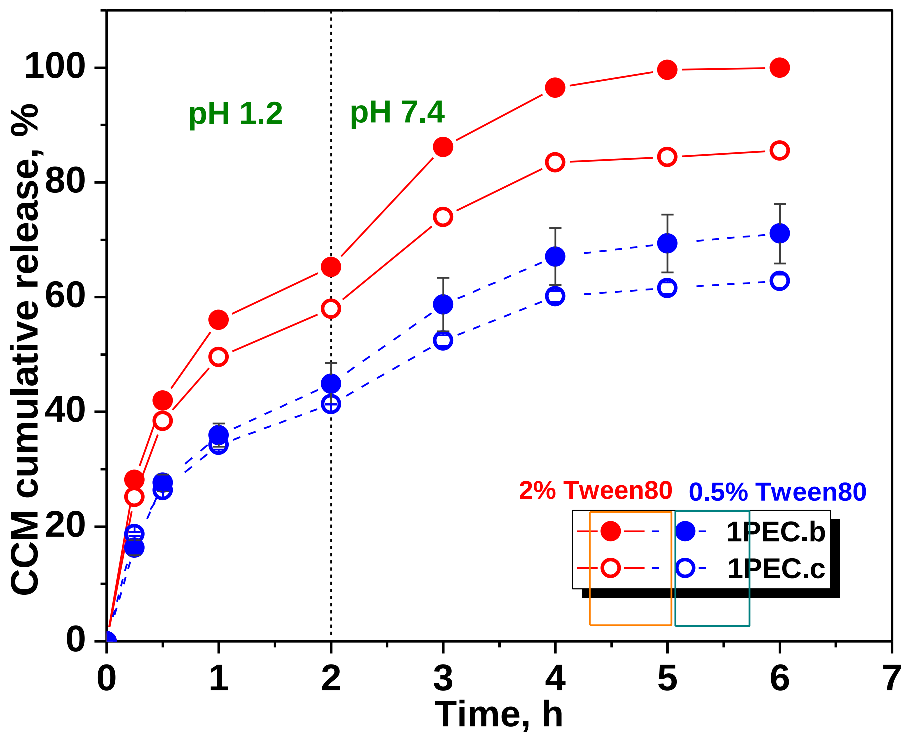

Figure S5. Cumulative release of CCM from the samples 1PEC.b and 1PEC.c (the same abbreviation as in Table 1). Loading of the samples was: 66.74 mg CCM/g 1PEC.b and 56.25 mg CCM/g 1PEC.c.
